# Exercise Exacerbates the Transcriptional Profile of Hypoxia, Oxidative Stress and Inflammation in Rats with Adjuvant-Induced Arthritis

**DOI:** 10.3390/cells8121493

**Published:** 2019-11-22

**Authors:** Susana Aideé González-Chávez, Celia María Quiñonez-Flores, Gerardo Pavel Espino-Solís, José Ángel Vázquez-Contreras, César Pacheco-Tena

**Affiliations:** 1 Facultad de Medicina y Ciencias Biomédicas; Universidad Autónoma de Chihuahua, Chihuahua 31109, Mexico; susana_glezch@hotmail.com (S.A.G.-C.); dr.cesarpacheco@gmail.com (C.P.-T.); gespinos@uach.mx (G.P.E.-S.); 2 Facultad de Ciencias de la Cultura Física; Universidad Autónoma de Chihuahua, Chihuahua 31109, Mexico; 3 Hospital Infantil de Especialidades de Chihuahua, Chihuahua 31090, Mexico

**Keywords:** running, exercise, physical activity, rheumatoid arthritis, adjuvant-induced arthritis

## Abstract

Physical exercise (PE) is recommended for Rheumatoid Arthritis (RA), but the molecular and biological mechanisms that impact the inflammatory process and joint destruction in RA remain unknown. The objective of this study was to evaluate the effect of PE on the histological and transcriptional changes in the joints of adjuvant-induced arthritis (AIA) rat model. AIA rats were subjected to PE on a treadmill for eight weeks. The joints were subjected to histological and microarray analysis. The differentially expressed genes (DEGs) by PE in the arthritic rats were obtained from the microarray. The bioinformatic analysis allowed the association of these genes in biological processes and signaling pathways. PE induced the differential expression of 719 genes. The DEGs were significantly associated with pathogenic mechanisms in RA, including HIF-1, VEGF, PI3-Akt, and Jak-STAT signaling pathways, as well as response to oxidative stress and inflammatory response. At a histological level, PE exacerbated joint inflammatory infiltrate and tissue destruction. The PE exacerbated the stressed joint environment aggravating the inflammatory process, the hypoxia, and the oxidative stress, conditions described as detrimental in the RA joints. Research on the effect of PE on the pathogenesis process of RA is still necessary for animal models and human.

## 1. Introduction

Rheumatoid arthritis (RA) is one of the most common inflammatory arthritis affecting 0.5–1.0% of the population [1]. RA is a systemic autoimmune disease characterized by inflammation of the synovial membrane and periarticular structures. Joint pain, stiffness, and swelling are the primary symptoms, which eventually lead to joint deformity and functional disability. Extra-articular manifestations, including cardiovascular, pulmonary, psychological and skeletal disorders are common in RA patients [2]. The etiology and pathophysiology of RA remain unresolved; however, genetic and environmental factors have been associated with the inappropriate immunomodulation that triggers the inflammatory process in the joints and the subsequent damage to synovial structures [3]. The treatment of RA aims to reduce pain and inflammation in the joints and also to preserve their structural integrity and the patient’s functionality. Currently, treatment includes a variety of pharmacological agents, education programs, joint protection, lifestyle changes, PE and surgical intervention as a final step [4,5,6].

PE is considered in the multidisciplinary management of RA patients. The European League Against Rheumatism (EULAR) has established the recommendations for physical activity in people with inflammatory arthritis [4], and its recommendations for cardiovascular disease risk management in RA include regular exercise [7]. It has been previously shown that PE improves joint health [8], muscle strength [9], cardiovascular fitness [10], vascular function [11], and psychological well being [12]. PE also reduces the inflammation [13], rheumatoid cachexia [14], and fatigue [15] in RA patients.

Although the systemic benefits of PE in rheumatic patients are well recognized, the direct consequences of PE in active synovitis and the potential role of PE accelerating joint destruction are not clear. Some reports have shown that mechanical load influences the onset and worsens the progression of the disease in experimental animal models of arthritis [16,17]; therefore, the consequences of PE on inflamed joints remain a topic that should be addressed.

We selected adjuvant-induced arthritis (AIA) because it mimics the signs and symptoms of human RA, such as the histopathological changes of inflammation, vascular proliferation, and importantly, the progression to joint destruction [18]. The AIA model has been widely used in the development of antirheumatic drugs, including several non-steroid anti-inflammatories, methotrexate [19], and also biologic drugs, including tocilizumab [20] and Jak-inhibitors like tofacitinib [21].

To understand the effects of PE in the complexity of the inflamed joint, we selected an unbiased approach to maintain a broad perspective. We selected the bioinformatic analysis of the transcriptome from the tarsal joint through a DNA microarray. The microarray simultaneously evaluates the expression levels for a large number of genes [22]; and, identifies the most relevant mediators in complex processes such as the interplay between PE and inflammation.

A better understanding of the molecular consequences of PE on the inflamed joints may contribute to improving its prescription for RA patients for the protection of joint integrity. In this regard, the objective of this study was to determine the effect of an exercise intervention on the transcriptional expression of genes in the AIA rat model using microarray technology.

## 2. Materials and Methods

### 2.1. Animals and Study Groups

Male Wistar rats (300–350g) were used for this study. The animals were housed in an animal facility with a 12 h light/dark cycle maintained at temperatures between 22 ± 1 °C with food and water provided at libitum. The experimental protocol was approved by the Committee of Ethics of the Instituto Chihuahuense de Salud-Secretaría de Salud-Facultad de Medicina y Ciencias Biomédicas, UACH (protocol number CEI-EXP-140/15). The animals were randomly divided into two groups of seven animals each: (1) arthritis group and (2) arthritis-exercise group.

### 2.2. Arthritis Induction

The AIA model was conducted as previously reported [23]. Rats were injected in the footpads with 0.2 mL of Complete Freund’s Adjuvant (CFA) (Sigma Chemicals, St. Louis, MO, USA) mixed with phosphate buffer saline in the 1:1 ratio. To increase the severity of arthritis, a booster injection with 0.1 mL of the emulsion was administered in the same way on day 5–post first injection.

### 2.3. Familiarization

Animals belonging to the arthritis-exercise group were familiarized with the treadmill for three weeks to reduce their stress level during the PE. Each daily familiarization session included placing the rats on the treadmill switched off for 10 min (visual/olfactory adaptation) and then turning on the treadmill at the minimum speed (3 m/min) for 5 min (sound/movement adaptation). After the first week of the treadmill familiarization, arthritis induction was started and the familiarization period continued for two more weeks.

### 2.4. Physical Capacity Test

After the familiarization, a physical capacity test (PCT) was conducted to establish the maximum speed reached by the animals before the intervention of PE. The PCT consisted of a single treadmill session in which the rats, after 5 min of warm-up (3 m/min), ran in the band with an increase in the speed of 3 m/min every 2 min until they fatigued and stopped running. The maximal physical capacity (100%) was defined as the maximum speed reached by each animal. The average speed per group was calculated, and this was used to establish the speed of the exercise sessions.

### 2.5. Exercise Program

The rats were exercised three times a week for eight weeks. The exercise started with a speed of 20% of the PCT and increases of 13% in speed were applied every two weeks until reaching 60%. Each exercise session included four phases: (1) acclimatization: rats were placed on the treadmill switched off for 5 min; (2) warm-up: rats walked for 5 min at lowest speed; (3) exercise: rats run for 25 min at the corresponding speed; and (4) cool down: rats walked at lowest speed for 5 min. The exercise sessions were administered at the same time each day.

### 2.6. Histological Analysis

After exercise intervention, the rats were euthanized, and the tarsal joints were immediately dissected. The ankle, the subtalar and the navicular cuboid joints were extracted and cut proximally in the distal fibula and tibia; and, distally in the metatarsal bones at the diaphysis. The muscle was dissected as much as possible and the synovial and ligament structures were preserved and fixed in 10% formalin phosphate buffer for 48 h and decalcified with 5% nitric acid for 24 h [24]. The tissues were dehydrated in graded ethanol, and embedded in paraffin, sectioned, and stained with hematoxylin-eosin (H&E Merck, Darmstadt, Germany). The histological variables: (a) inflammatory infiltrate, (b) synovial hyperplasia, (c) pannus formation, (d) synovial vascularity, (e) cartilage damage and (f) bone erotion were semi-quantitatively evaluated using a scale of 0 = absent, 1 = mild, 2 = moderate and, 3 = severe on each animal. The maximum score reached per animal for each parameter was 12 when the four joints showed a severe level. The scoring average per study group was estimated. The histological variables were evaluated bu two experienced operators blinded to the different groups.

### 2.7. DNA Microarray and Bioinformatics Analysis

The effect of PE on transcriptome was evaluated in the DNA microarray by comparing the arthritis-exercise group (experimental group) and the arthritis group (reference group). The tarsal joints dissected were immediately placed in liquid nitrogen and disrupted with mortar and pestle. Total RNA was extracted using RNeasy^®^ Lipid Tissue Mini Kit (QIAGEN, Germantown, MD, USA) according to the manufacturer´s instructions. The RNA quality and integrity were verified using a 2100 Bioanalyzer (Agilent Technologies, Santa Clara, CA, USA). For the microarray, the RNA of every group was pooled keeping equimolar quantities of every individual.

The microarray analysis was performed at the Institute of Cellular Physiology of the National Autonomous University of Mexico (México City, México). Briefly, the cDNA was synthesized and labeled for subsequent hybridization in the Rn5K microchip containing 5000 rat genes. Scanning and signal acquisition was performed using the ScanArray 4000 (Packard BioChips Technologies, Billerica, MA, USA). The GenArise software was used for the analysis of the microarray scan, and the lists of DEGs [Z-score ≥ 1.5 standard deviations (SD)] were obtained. To assess the biological relevance of the DEGs, DAVID Bioinformatics Resources 6.8 platform (https://david.ncifcrf.gov/) was used. Gene Ontology (GO) and KEGG (Kyoto Encyclopedia of Genes and Genomes) pathway mapper with significant associations (*p* ≤ 0.5) were obtained [25]. Also, the STRING 10.5 database (http://string-db.org/) was used to obtain the analysis and integration of direct and indirect protein-protein interactions (IPP) centered on the functional association [26]. The DEGs identified in the microarray were loaded, and the interactions were selected with minimal confidence (interaction score > 0.4). The obtained IPP network was analyzed more thoroughly to obtain primary clusters of sub-networks using the Cytoscape software version 3.7.0 (Bethesda, Rockville, MD, USA) with the Molecular Complex Detection (MCODE) complement [27,28].

### 2.8. Statistical Analysis

The statistical analysis was made in SPSS statistics v22 software (SPSS Science Inc., Chicago, IL, USA). Measures of central tendency were estimated for each variable. T-test was used to compare the effect of PE on histological parameters. Differences were considered significant when *p* < 0.05.

## 3. Results

### 3.1. Maximal Physical Capacity

The maximal physical capacity obtained in the PCT for the arthritis-exercise group was 21.28 ± 5.96 m/min. The initial speed and its increments along the exercise intervention were based on this value.

### 3.2. Histological Analysis

**Figure 1:**
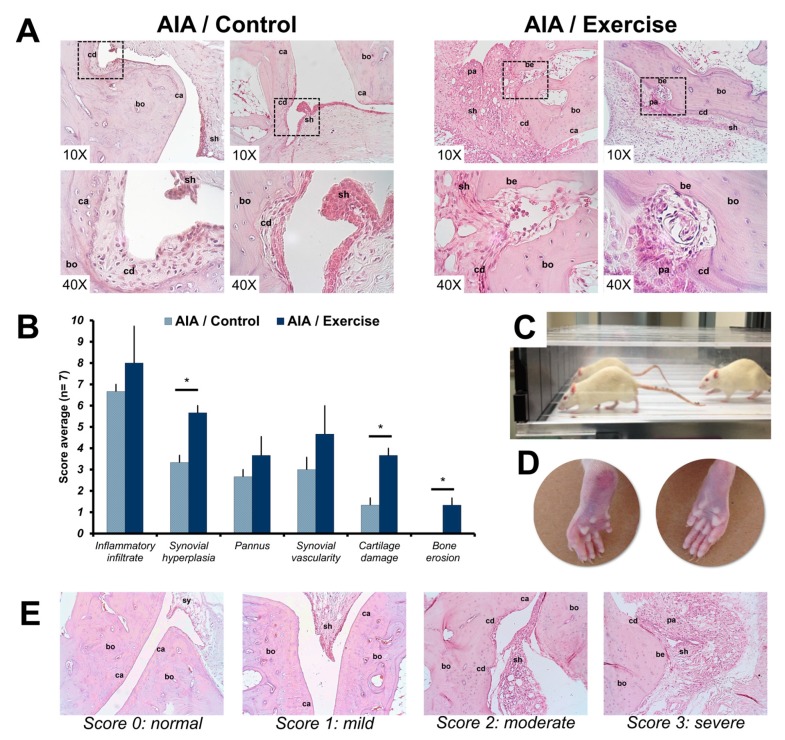
Effects of exercise on tarsal bone histological parameters in adjuvant-induced arthritis rats. (**A**) Representative images of histological findings in the tarsal joints of the study groups at the end of the exercise intervention using H&E staining. (**B**) Joint involvement was scored by the semi-quantitative scale (showed in E) to describe inflammatory changes and structural remodeling in the tarsal joints (7 rats per group). The t-student test was used to compare histological measurements between groups. * *p* < 0.01. (**C**) Exercised rats on a treadmill. (**D**) Representative images of clinical changes on hind paws of adjuvant-induced arthritis (AIA) rats exercised (left) and non-exercised (right). (**E**) Representative images of the inflammation and structural joint damage scores in the tarsal joints of AIA rats. The 0 (normal) score was established in healthy rats, where the bone (bo), cartilage (ca) and synovium (sy) did not show alterations. The arthritis scores 1 (mild), 2 (moderate), and 3 (severe) were based on the inflammatory changes: the presence of synovial hyperplasia (sh) and pannus (pa) and structural remodeling: cartilage damage (cd) and bone erosion (be). The images were acquired with a 10× and 40× amplification. AIA: adjuvant-induced arthritis.

The effect of exercise in the joints was histologically evaluated using H&E staining. The scores of inflammatory infiltrate, synovial hyperplasia, pannus formation, synovial vascularity, and cartilage and erosions obtained for each study group are shown in Figure 1. The highest scores of all evaluated parameters were reached in the arthritis exercise group. However, only hyperplasia, cartilage damage and bone erosion were statistically different between the groups.

### 3.3. Microarray and Bioinformatic Analysis

The transcriptome-wide microarray analysis identified a pool of DEGs in the joints from arthritic exercised rats in comparison to arthritic non-exercised rats. A total of 719 genes were differentially expressed (Z-score ≥ 1.5 SD), 361 up-regulated (Appendix A), and 358 down-regulated (Appendix B). AB000928 (Zp1) and X68101 (Trg) were the most significantly up- and down-expressed genes by PE, respectively.

**Figure 2:**
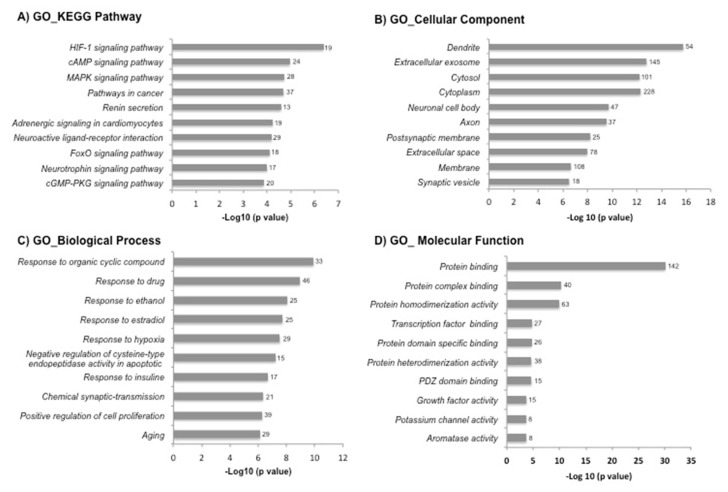
The Gene Ontology (GO) and Kyoto Encyclopedia of Genes and Genomes (KEGG) pathway enrichment analysis of differentially expressed genes in the DAVID database. The 719 differentially expressed genes by exercise were uploaded into the DAVID database for enrichment analysis. The top 10 GO analysis results of these dysregulated genes were displayed in the bar chart: (**A**) KEGG pathway, (**B**) cellular component, (**C**) biological process and (**D**) molecular function. The bars indicated the -Log10 (*p* value) of each GO and KEGG term. The number of genes involved in each term is shown on the right side of each bar.

To understand the related biological functions of the 719 DEGs, we used the DAVID database for the enrichment analysis of GO and KEGG pathways. The top 10 enriched terms of GO and KEGG pathways are shown in Figure 2. Response to organic cyclic compound, response to drug, and response to ethanol were among the top enriched biological processes. The DEGs were enriched in molecular functions like protein binding. The most enriched KEGG pathways included hypoxia-induced factor-1 (HIF-1), cyclic adenosine monophosphate (cAMP) and mitogen-activated protein kinase (MAPK) signaling pathways. Subsequently, we selected the biological processes and KEGG signaling pathways known to be relevant in the pathogenesis of RA, which included: response to hypoxia, response to oxidative stress, angiogenesis and inflammatory/immune response (Table 1).

**Figure 3:**
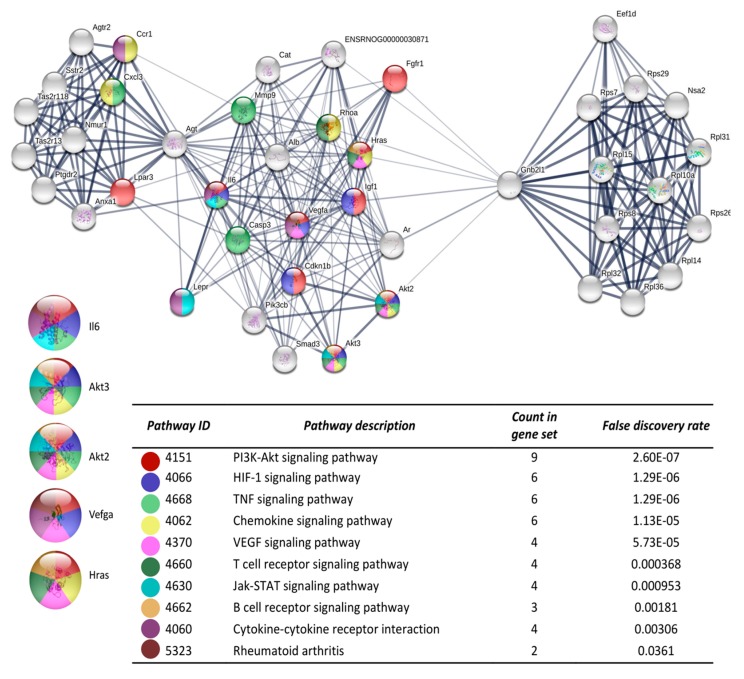
The protein-protein interaction network construction of the cluster number one obtained with the differentially expressed genes. The lists of the differentially expressed genes (Z-score ≥ 1.5 SD) were analyzed on the STRING and Cytoscape platforms. The primary clusters of sub-networks were obtained using the Molecular Complex Detection (MCODE) complement (cutoff = 0.2). Line thickness indicates the strength of data support; colored nodes indicate query proteins and first shell of interactors; white nodes indicate the second shell of interactors.

**Figure 4:**
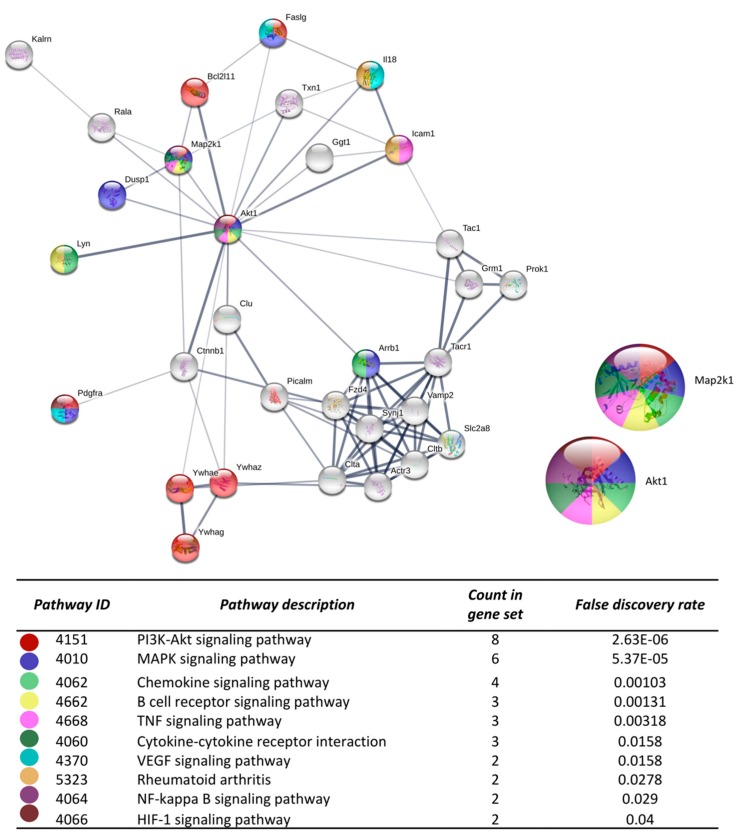
The protein-protein interaction network construction of the cluster number two obtained with the differentially expressed genes. The lists of the differentially expressed genes (Z-score ≥ 1.5 SD) were analyzed on the STRING and Cytoscape platforms. The primary clusters of sub-networks were obtained using the Molecular Complex Detection (MCODE) complement (cutoff = 0.2). Line thickness indicates the strength of data support; colored nodes indicate query proteins and first shell of interactors; white nodes indicate the second shell of interactors.

**Figure 5:**
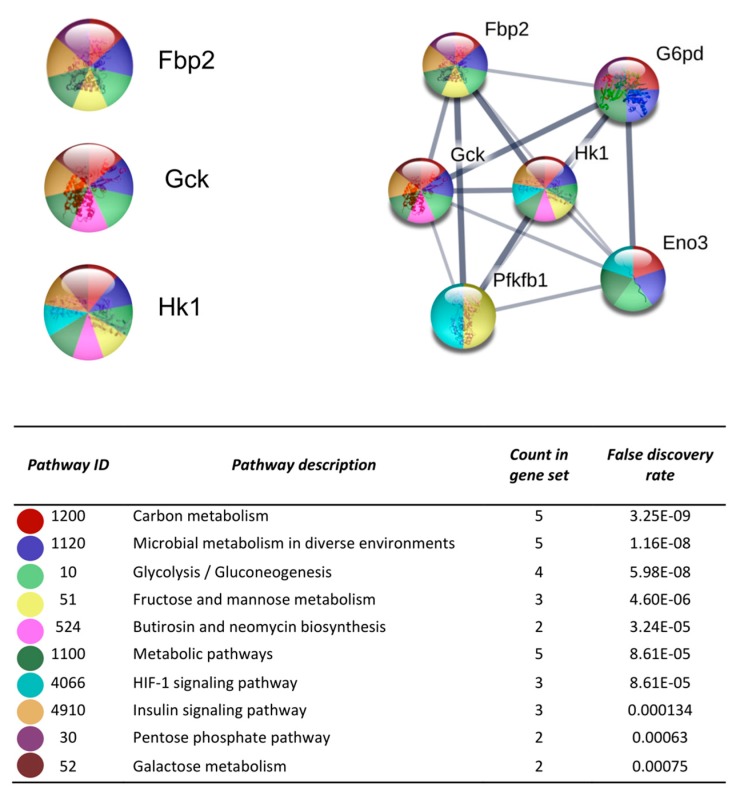
The protein-protein interaction network construction of the cluster number three obtained with the differentially expressed genes. The lists of the differentially expressed genes (Z-score ≥ 1.5 SD) were analyzed on the STRING and Cytoscape platforms. The primary clusters of sub-networks were obtained using the Molecular Complex Detection (MCODE) complement (cutoff = 0.2). Line thickness indicates the strength of data support; colored nodes indicate query proteins and first shell of interactors; white nodes indicate the second shell of interactors.

After the analysis on DAVID, the list of the 719 DEGs was analyzed on the STRING and Cytoscape-MCODE platforms. The first three clusters obtained are shown in Figure 3, Figure 4 and Figure 5. The genes from the first three clusters were loaded in STRING to identify the associated KEGG signaling pathways; those relevant in RA were selected and marked with different colors. The pathways considered as relevant included the HIF-1 signaling pathway. The analysis of the protein networks unveiled relevant genes dysregulated by PE. Those presented multiple functional connections included up-regulated genes such as serine-threonine protein kinases Akt1, Akt2, and Akt3; the vascular endothelial growth factor A (Vefga), the mitogen-activated protein kinase kinase 1 (Mapk1), phosphatidylinositol 4,5-bisphosphate 3-kinase catalytic subunit beta isoform (Pik3cb), glucokinase (Gck), fructose-biphosphatase2 (Fbp2); and also down-regulated genes such as interleukin 6 (Il6).

## 4. Discussion

Current evidence describing the influence of PE on the pathogenic mechanisms of the arthritides is scarce and limited to studies in animal models. Most of these studies have focused on animal models of osteoarthritis and, in most cases, only clinical and histological parameters have been evaluated, limiting our understanding of the PE in the pathogenesis of active synovitis. To our knowledge, no previous study has assessed the effects of PE on the transcriptome profile in the AIA model.

Our study shows that PE induced several genes linked to RA pathogenesis. Specifically, PE exacerbated hypoxia, oxidative stress, and the immune/inflammatory response in rats with AIA. The histology confirms our transcriptome results; in the joints of the exercised animals, we observed an increase in the inflammatory infiltrate and more severe scores for bone and cartilage destruction.

In our opinion, the connection between PE and its potential detrimental role at active stages of synovitis should be considered as a relevant event in patients with inflammatory arthritides. Current recommendations for physical activity and PE in people with inflammatory and non-inflammatory arthritis [4,5,7,29] have been established mostly on clinical studies in humans, which the intimate effect of PE in the arthritic joints at a molecular level has not been considered. These recommendations have not been specified according to the disease activity; however, as our study shows, likely at very active stages of inflammatory arthritides, PE could increase the inflammatory process. It is unlikely that PE will be prescribed in patients with active arthritis; however, a wide prescription of PE could also include the large proportion of patients whom remain with some degree of inflammatory activity despite therapy. Probably, the PE benefits may not be as clear in those patients with partial disease control. Therefore, the disease activity should be considered as an element for PE prescription.

RA pathogenesis remains a primary research field; we now understand that the rheumatoid pannus is an altered microenvironment that carries a significant array of abnormal immune processes and also several metabolic alterations. The increase in hypoxia and oxidative stress are recognized as relevant inflammation drivers within the rheumatic synovium. Their pathological implications are now sustained by experimental results both: in animal models [30,31] and human disease [32,33]. Synovial hypoxia promotes inflammation, angiogenesis, production of chemokines, and cartilage and bone destruction, mediated in part, through HIF-1 that is a potent pleiotropic mediator [32,34].

Our study confirms that PE up-regulates genes linked to hypoxia and oxidative stress in the arthritic joints; indeed, the HIF-1 signaling was the first enriched pathway in our bioinformatic analysis, suggesting that this pathway was the strongest influenced by the PE. Downstream HIF-linked genes as the vascular endothelial growth factor (VEGF) are also up-regulated. VEGF is critical for the onset and perpetuation of vascular proliferation in synovitis [35,36,37]. Also, it was previously reported the VEGF increase in the serum and synovial fluid of RA patients [35,38] as well as in experimental animal models [39].

Hypoxia could activate the genes encoding nearly every step of glycolysis [32]. Abnormal glucose metabolism was observed in the synovial fluid and synovium of RA, which is evidenced by increased glycolytic enzyme activity [40]. A recent study proposes the inhibition of hexokinases as a potential therapeutic strategy in the treatment of patients with RA [41]. According to this, the glucokinase (Gck) and the hexokinase 1 (Hk1) were up-regulated by PE in our study. Additionally, genes involved in the oxidative stress process were also dysregulated by PE. It is recognized that in an inflamed joint, hypoxia and reperfusion cycles occur, producing an excessive amount of reactive oxygen species (ROS) known as oxidative stress [33,42].

In addition to hypoxia and oxidative stress conditions induced by the effect of PE, other KEGG pathways were dysregulated (Table 1) such as rheumatoid arthritis, T-cell and B-cell receptor signaling, chemokine, Jak-Stat, TNF, Toll-like-receptor, WNT and osteoclast differentiation signaling pathways. This array of dysregulated pathways suggests a widespread effect of PE both in the adaptive and innate immune response.

Other genes participating in several KEGG pathways in our study include Akt1, Akt2, Akt3, Map2k1 and PIk3cb which belong to PI3K/AKT/mTOR and MAPK pathways that are considered central in RA pathogenesis [43,44]. They have been implicated in fibroblast-like synoviocyte proliferation and activation. They stimulate the production of pro-inflammatory and osteoclastogenic cytokines; besides, the inhibition of either pathway has been correlated with the improvement of disease activity parameters. Therefore, the use of agents with the potential to regulate either PI3K/AKT/mTOR or MAPK pathways has been considered as a potential therapeutic strategy [45].

The molecular results of the PE effect are not easily comparable with previous studies due to the low number of reports, which had objectives aimed at particular aspects of the disease. However, we can distinguish that the negative effect of PE has also been reported in the two most recent and complete studies in this field [16,17]. The results of these studies suggest a direct link between the degree of mechanical stress to inflammation and tissue localization, which strengthens previous studies that propose biomechanical factors as potential determinants for the topographic pattern of joint disorders in arthritis [46]. Cambré et al., suggest that the link between mechanical stress and the onset of arthritis is explained by local recruitment of Ly6high inflammatory cells elicited by mechanostress-induced chemokine induction in resident mesenchyme cells [17]. They also provide evidence of the local participation of the complement activators to maintain the progression and chronicity of arthritis through an impaired resolution [16].

On the other hand, it has been shown that in healthy rats, PE has a positive effect in the joints it induces extracellular matrix biosynthesis, cartilage strengthening, and attenuation of inflammatory pathways [47]. This suggests that the gene induction pattern of mechanosensitive genes seems to be distinct between different arthritis models and healthy animals, and likely also in regarding to the degree of arthritis activity.

Consistent with the genetic analysis, our histological findings showed that PE had an adverse effect on increasing the inflammatory infiltrate and the joint destruction. In experimental models of arthritis, it has been confirmed that mechanical stress increase joint inflammation, stimulate the progression and chronicity of arthritis and structural damage [16,17,48]; besides, the mechanical stress decrease the bone quality [16]. In contrast to our findings, other studies in the AIA model have shown an anti-inflammatory effect of PE in some parameters such as synovial hyperplasia [49,50], and joint destruction [51].

Mode, intensity, frequency, timing, and duration of the PE protocol are decisive aspects to the physiological responses and different outcomes [52]. Moreover, the severity of arthritis at the starting time of PE could be definitory. Studies in the AIA model that started the PE at the pre-arthritic stage [49,50] showed beneficial results. However, we started the PE at an arthritic stage because we think this scenario is more representative of RA patients.

## 5. Conclusions

The present study explored the influence of PE in the genetic expression and the histology of the actively swollen joints in the AIA model. Our results describe an exploratory and preliminary scenario in which PE increased the expression of genes with a known pathogenic role in RA; these findings were consistent with the histological findings. These results suggest that the PE exacerbated the stressed joint environment, where the inflammation, hypoxia, and the oxidative stress prevail, rendering clear parallels to the RA joints. The molecular effect of exercise on active stages of inflammatory arthropathies requires further studies in animal models and humans, which could contribute with the development of adequate programs for RA patients that can ensure a beneficial effect without negative implications at the tissue level.

## Figures and Tables

**Table 1:** Differentially expressed genes by physical exercise associated with pathogenic processes in Rheumatoid Arthritis.

Description		Effect on Regulation	Number of Genes	Genes	*p*-Value
***Biological Process***	Response to hypoxia	Up	12	Camk2d, Cat, Cdkn1b, Il18, Lepr, Mt3, Nppa, Ptk2b, Rhoa, Tgfbr3, Vegfa, Vhl	3.1 × 10^−3^
		Down	17	Alas1, Bnip3, Cited2, Ep300, Smad3, Casp3, Hmbs, Hyou1, Igf1, Itga2, Icam1, Il1a, Mmp9, Kcnma1, Th, Ucp2, Ucp3	7.5 × 10^−6^
	Oxidation-reduction process	Up	19	Akr1a1, Aifm1, Cbr1, Cyp2d2, Cyp2t1, Cyp27b1, Cyp4f6, Cyp51, Degs1, Gpx1, Hao2, Ido1, Kif1b, Me1, Oxr1, Prdx2, Pah, Txn1, Tpo	1.2 × 10^−2^
		Down	26	Haao, Hibadh, Ndufa12, Aldh6a1, Aox1, Cp, Crym, Cyp19a1, Cyp1b1, Cyp2a3, Cyp2b12, Cyp2p2c7, Cyp2d3, Dio2, Dcxr, G6pd, Glrx, Gpx6, Hsd3b6, Hadhb, Ldhb, Scd, Srd5a2, Suox, Tpo, Th	4.9 × 10^−5^
	Hydrogen peroxide catabolic process	Up	4	Cat, Gpx1, Prdx2, Tpo	2.5 × 10^−3^
	Response to oxidative stress	Up	8	Akt1, Cat, Gpx1, Mt3, Map2k1, Oxr1, Prdx2, Tpo	7.4 × 10^−3^
	Cellular response to oxidative stress	Up	5	Ggt1, Mt3, Nfe2l2, Prdx2, Txn1	3.2 × 10^−2^
	Cellular response to hydrogen peroxide	Up	5	Anxa1, Aifm1, Il18, Nfe2l2, Ppp5c	3.5 × 10^−2^
	Response to reactive oxygen species	Up	3	Cat, Gpx1, Ptk2b	4.1 × 10^−2^
	Inflammatory response	Up	12	Akt1, Ccl4, Anxa1, Cxcl3, Csf1, Cyp4f6, Crlf2, Hmgb1, Ido1, Il18, Mep1b, Nfe2l2	6.3 × 10^−3^
***KEGG signaling pathway***	HIF-1 signaling pathway	Up	14	Akt1, Akt3, Camk2a, Camk2d, Cdkn1b, Hk1, Ifngr1, Map2k1, Nppa, Pik3cb, Pik3r1, Tceb2, Vegfa, Vhl	2.5 × 10^−7^
	VEGF signaling pathway	Up	8	Akt1, Akt3, Map2k1, Pik3cb, Pik3r1, Ppp3cb, Ppp3r2, Vegfa	2.5 × 10^−4^
	Rheumatoid Arthritis	Up	6	Atp6v0a1, Atp6v0e1, Cd80, Csf1, Il18, Vegfa	4.2 × 10^−2^
	T cell receptor signaling pathway	Up	10	Akt1, Akt3, Dlg1, Lcp2, Map2k1, Pik3cb, Pik3r1, Ppp3cb, Ppp3r2, Rhoa	4.5 × 10^−4^
	B cell receptor signaling pathway	Up	7	Akt1, Akt3, Map2k1, Pik3cb, Pik3r1, Ppp3cb, Ppp3r2	3.3 × 10^−3^
	Chemokine signaling pathway	Up	12	Akt1, Akt3, Ccl4, Gng8, Adcy5, Arrb1, Cxcl3, Map2k1, Pik3cb, Pik3r1, Ptk2b, Rhoa	1.3 × 10^−3^
	PI3K-Akt signaling pathway	Up	17	Akt1, Akt3, Faslg, Gng8, Csf1, Cdkn1b, Fgd17, Fgf19, Il3ra, Lpar3, Map2k1, Pik3cb, Pik3r1, Ppp2r2c, Vegfa, Ywhae, Ywhag	2.2 × 10^−3^
	Jak-STAT signaling pathway	Up	10	Akt1, Akt3, Cish, Crlf2, Ifngr1, Il3ra, Lepr, Pik3cb, Pik3r1, Thpo	2.2 × 10^−3^
	TNF signaling pathway	Up	7	Akt1, Akt3, Cxcl3, Csf1, Map2k1, Pik3cb, Pik3rc1	2.8 × 10^−2^
	Toll-like receptor signaling pathway	Up	7	Akt1, Akt3, Ccl4, Cd80, Map2k1, Pik3cb, Pik3r1	1.7 × 10^−2^
	Wnt signaling pathway	Up	10	Wif1, Camk2a, Camk2d, Csnk2a1, Csnk2b, Ctnnb1, Fzd4, Ppp3cb, Ppp3r2, Rhoa	3.0 × 10^−3^
	Osteoclast differentiation	Up	13	Akt1, Akt3, Fcgr2a, Csfr1, Ifngr1,Lilrb3l, Lcp2, Mapk2k1, Pik3cb, Pik3r1, Ppp3cb, Ppp3r1, Ppp3r2	8.6 × 10^−5^

## Appendix A. List of Genes Up-Regulated by PE Effect

**Gene Id**	**Gene**	**Z-Score**
AA818786	No data	1.770223
AA963507	No data	1.500387
AB000928	zona pellucida glycoprotein 1(Zp1)	3.330347
AB003753	No data	2.348897
AB012139	bone morphogenetic protein 1(Bmp1)	1.717532
AB022014	proteasome 26S subunit, non-ATPase 10(Psmd10)	1.800734
AB024333	barrier to autointegration factor 1(Banf1)	2.409521
AB027143	septin 5(Sept5)	2.332071
AB037248	ATPase H^+^ transporting V0 subunit e1(Atp6v0e1)	1.790973
AB041723	apoptosis inducing factor, mitochondria associated 1(Aifm1)	2.20147
AB043959	ubiquitin C-terminal hydrolase L3(Uchl3)	2.083474
AB070355	kinesin family member 1B(Kif1b)	2.560659
AB073318	brain and acute leukemia, cytoplasmic(Baalc)	1.825273
AF000139	cytochrome P450, family 27, subfamily b, polypeptide 1(Cyp27b1)	3.142163
AF016183	vomeronasal 2 receptor, pseudogene 45(Vom2r-ps45)	2.284991
AF026554	solute carrier family 5 member 6(Slc5a6)	1.552609
AF030243	interleukin 3 receptor subunit alpha(Il3ra)	1.890042
AF041374	phosphatidylinositol binding clathrin assembly protein(Picalm)	2.019124
AF053989	vomeronasal 2 receptor 44(Vom2r44)	1.534172
AF059678	replication factor C subunit 1(Rfc1)	1.961506
AF063103	adhesion G protein-coupled receptor L3(Adgrl3)	1.510015
AF063890	protein tyrosine kinase 2 beta(Ptk2b)	2.392998
AF065161	cytokine inducible SH2-containing protein(Cish)	2.074343
AF102262	beta-1,4-galactosyltransferase 1(B4galt1)	1.819501
AF106657	ubiquitin specific peptidase 15(Usp15)	2.061666
AF115768	defensin alpha 24(Defa24)	2.430658
AF121265	catenin beta 1(Ctnnb1)	1.995608
AF121893	single stranded DNA binding protein 3(Ssbp3)	1.660423
AF145050	eukaryotic translation elongation factor 1 delta(Eef1d)	1.902054
AF146738	centrosomal protein 19(Cep19)	1.815362
AF155196	No data	1.575313
AF169390	phosphodiesterase 6H(Pde6h)	1.774068
AF169636	leukocyte immunoglobulin-like receptor, subfamily B (with TM and ITIM domains), member 3-like(Lilrb3l)	3.098234
AF173834	calpain 3(Capn3)	1.680331
AF176023	PR/SET domain 4(Prdm4)	2.242755
AF178689	carbohydrate sulfotransferase 3(Chst3)	1.70415
AF187323	cathepsin Q(Ctsq)	2.106614
AF190256	phosphate cytidylyltransferase 1, choline, beta(Pcyt1b)	2.363304
AF201901	interferon gamma receptor 1(Ifngr1)	1.542342
AF208125	acyl-CoA synthetase bubblegum family member 1(Acsbg1)	2.403616
AF214647	N-acylsphingosine amidohydrolase 1(Asah1)	1.539499
AF218575	nibrin(Nbn)	1.875441
AF228043	nuclear receptor coactivator 6(Ncoa6)	1.721794
AF228917	zinc finger, DHHC-type containing 2(Zdhhc2)	1.609594
AF237778	calcium/calmodulin-dependent protein kinase II alpha(Camk2a)	1.638852
AF273025	solute carrier family 38, member 3(Slc38a3)	2.373408
AF276940	ectonucleoside triphosphate diphosphohydrolase 2(Entpd2)	2.0331
AF281304	potassium channel, two pore domain subfamily K, member 6(Kcnk6)	2.029054
AF288611	RUN domain containing 3A(Rundc3a)	2.840557
AF303035	PARP1 binding protein(Parpbp)	2.933101
AF308818	KH-type splicing regulatory protein(Khsrp)	1.694262
AF311055	thioredoxin 1(Txn1)	2.848762
AF333325	HPS1, biogenesis of lysosomal organelles complex 3 subunit 1(Hps1)	1.833169
AF333986	oxidation resistance 1(Oxr1)	2.393664
AF336113	actin-binding Rho activating protein(Abra)	2.216764
AF345444	potassium voltage-gated channel interacting protein 4(Kcnip4)	2.743564
AF368269	cytochrome P450, family 2, subfamily t, polypeptide 1(Cyp2t1)	1.620044
AF393750	BPI fold containing family A, member 1(Bpifa1)	2.365618
AF398465	dihydropyrimidinase-like 3(Dpysl3)	1.872371
AF419333	gamma-aminobutyric acid type A receptor theta subunit(Gabrq)	1.792286
AI059116	No data	1.626247
AI102932	No data	1.930485
AI104638	No data	2.595373
AI105022	No data	1.646591
AI113337	No data	1.994172
AI230498	No data	1.568518
AI230682	No data	2.036962
AI231775	No data	1.560267
AI385377	No data	3.220256
AI406694	No data	2.313044
AI500802	No data	2.504556
AI535093	No data	1.900459
AI599423	No data	1.514733
AI602844	No data	1.652924
AI717432	No data	1.677302
AJ012482	phosphatidylinositol-4,5-bisphosphate 3-kinase, catalytic subunit beta(Pik3cb)	2.039374
AT005481	No data	1.678314
AW141757	No data	1.778821
AW520335	No data	2.449793
AW533345	No data	1.75804
AW914284	No data	1.704328
AW915815	No data	1.91521
AW916410	No data	1.858779
AW918157	No data	1.600146
AW918457	No data	1.504258
AW918850	No data	1.940086
AW921253	No data	1.848394
AY026068	ras homolog family member A(Rhoa)	2.033377
AY030278	Wnt inhibitory factor 1(Wif1)	1.764736
AY034383	dynein light chain LC8-type 2(Dynll2)	1.527297
AY035403	kinesin family member 6(Kif6)	1.9322
AY122322	protein phosphatase 1, regulatory (inhibitor) subunit 14D(Ppp1r14d)	2.374861
BE100543	No data	2.356497
BE113282	No data	2.311542
BE113420	No data	1.660364
BE118455	No data	1.925824
BF390636	No data	1.846075
BF405195	No data	1.559474
BF406553	No data	1.625267
BF411273	No data	1.537562
BF418537	No data	1.791187
BF420605	No data	1.918056
BF522262	No data	2.816838
BF522647	No data	1.689307
BF525282	No data	2.164385
BF543281	No data	1.772391
BF543321	No data	1.81299
BF546465	No data	2.021733
BF553424	No data	1.799168
BF561374	No data	1.753152
BF563877	No data	2.224055
BF564018	No data	1.608414
BF565044	No data	1.715644
BF565565	No data	1.534809
BG374035	No data	1.848573
BG664137	No data	1.654493
BG665039	No data	1.996679
BG666176	No data	2.409512
BG668317	No data	1.503399
BG668660	No data	1.902199
BG671436	No data	2.520046
BG671873	No data	2.019116
BG673258	No data	2.563218
BI282584	No data	2.135856
BM383757	No data	1.584869
BQ207374	No data	1.532423
BQ211469	No data	1.62545
BU671396	No data	1.554288
BU671701	No data	2.802387
D00729	enoyl-CoA delta isomerase 1(Eci1)	2.814065
D14480	calpain 8(Capn8)	1.674437
D21095	chemokine (C-X-C motif) ligand 3(Cxcl3)	1.92518
D30040	AKT serine/threonine kinase 1(Akt1)	1.680116
D38261	protein phosphatase 2, regulatory subunit B, gamma(Ppp2r2c)	1.79365
D85435	protein kinase C, delta binding protein(Prkcdbp)	1.78163
D87950	ubiquilin 1(Ubqln1)	1.763773
J00744	No data	1.516659
L04760	tripartite motif-containing 23(Trim23)	2.018875
L07315	dipeptidase 1 (renal)(Dpep1)	1.601127
L13606	myosin heavy chain 2(Myh2)	1.787821
L15618	casein kinase 2 alpha 1(Csnk2a1)	1.7187
L28801	general transcription factor IIIC subunit 1(Gtf3c1)	1.516161
L29427	No data	2.009211
L35921	G protein subunit gamma 8(Gng8)	1.713815
M13011	No data	1.614206
M15883	clathrin, light chain B(Cltb)	1.806758
M21759	filaggrin(Flg)	1.813956
M22030	No data	1.780357
M32062	Fc fragment of IgG, low affinity IIa, receptor(Fcgr2a)	1.60304
M33821	gamma-glutamyltransferase 1(Ggt1)	2.263792
M91450	No data	1.564184
M96626	ATPase plasma membrane Ca^2+^ transporting 3(Atp2b3)	2.172804
M97255	trefoil factor 2(Tff2)	1.647276
NM_012519	calcium/calmodulin-dependent protein kinase II delta(Camk2d)	1.505653
NM_012520	catalase(Cat)	2.274713
NM_012526	chromogranin B(Chgb)	2.018038
NM_012528	cholinergic receptor nicotinic beta 1 subunit(Chrnb1)	1.680012
NM_012531	catechol-O-methyltransferase(Comt)	2.231328
NM_012543	D-box binding PAR bZIP transcription factor(Dbp)	1.532421
NM_012549	endothelin 2(Edn2)	1.830311
NM_012565	glucokinase(Gck)	3.003472
NM_012572	glutamate ionotropic receptor kainate type subunit 4(Grik4)	1.960676
NM_012594	lactalbumin, alpha(Lalba)	2.35724
NM_012596	leptin receptor(Lepr)	1.572461
NM_012600	malic enzyme 1(Me1)	1.705429
NM_012612	natriuretic peptide A(Nppa)	1.752513
NM_012619	phenylalanine hydroxylase(Pah)	1.861168
NM_012653	solute carrier family 9 member A2(Slc9a2)	1.680892
NM_012663	vesicle-associated membrane protein 2(Vamp2)	1.751666
NM_012667	tachykinin receptor 1(Tacr1)	2.182398
NM_012695	sulfotransferase family 2A, dehydroepiandrosterone (DHEA)-preferring, member 6(Sult2a6)	1.605457
NM_012730	cytochrome P450, family 2, subfamily d, polypeptide 2(Cyp2d2)	1.829295
NM_012734	hexokinase 1(Hk1)	1.709348
NM_012788	discs large MAGUK scaffold protein 1(Dlg1)	1.700899
NM_012850	growth hormone releasing hormone receptor(Ghrhr)	1.557048
NM_012876	ribosomal protein S29(Rps29)	2.638145
NM_012904	annexin A1(Anxa1)	1.502437
NM_012908	Fas ligand(Faslg)	2.142956
NM_012910	arrestin, beta 1(Arrb1)	1.672619
NM_012941	cytochrome P450, family 51(Cyp51)	1.657199
NM_012963	high mobility group box 1(Hmgb1)	1.666837
NM_012978	luteinizing hormone/choriogonadotropin receptor(Lhcgr)	3.050579
NM_012980	matrix metallopeptidase 11(Mmp11)	2.630092
NM_012993	nardilysin convertase(Nrdc)	2.298098
NM_013005	phosphoinositide-3-kinase regulatory subunit 1(Pik3r1)	2.802073
NM_013008	POU class 1 homeobox 1(Pou1f1)	1.552156
NM_013030	solute carrier family 34 member 1(Slc34a1)	1.569844
NM_013183	meprin A subunit beta(Mep1b)	1.901411
NM_013224	ribosomal protein S26(Rps26)	1.620632
NM_013226	ribosomal protein L32(Rpl32)	2.284438
NM_013413	relaxin 1(Rln1)	1.971464
NM_017015	glucuronidase, beta(Gusb)	1.8737
NM_017042	protein phosphatase 3 catalytic subunit beta(Ppp3cb)	1.989406
NM_017137	chloride channel, voltage-sensitive 2(Clcn2)	1.867684
NM_017144	troponin I3, cardiac type(Tnni3)	1.571609
NM_017169	peroxiredoxin 2(Prdx2)	2.126629
NM_017193	aminoadipate aminotransferase(Aadat)	2.633207
NM_017251	gap junction protein, beta 1(Gjb1)	1.77218
NM_017256	transforming growth factor beta receptor 3(Tgfbr3)	1.864647
NM_017264	proteasome activator subunit 1(Psme1)	1.819425
NM_017279	proteasome subunit alpha 2(Psma2)	1.725385
NM_017348	solute carrier family 6 member 8(Slc6a8)	1.628447
NM_019137	early growth response 4(Egr4)	1.894886
NM_019151	myostatin(Mstn)	2.351448
NM_019161	cadherin 22(Cdh22)	2.206278
NM_019165	interleukin 18(Il18)	1.52836
NM_019170	carbonyl reductase 1(Cbr1)	1.714897
NM_019179	thymidylate synthetase(Tyms)	1.837275
NM_019198	fibroblast growth factor 17(Fgf17)	2.35561
NM_019216	growth differentiation factor 15(Gdf15)	1.772315
NM_019266	sodium voltage-gated channel alpha subunit 8(Scn8a)	2.082371
NM_019272	ssemaphorin 4F(Sema4f)	1.685348
NM_019277	exocyst complex component 6(Exoc6)	1.957294
NM_019291	carbonic anhydrase 2(Car2)	2.185707
NM_019323	mast cell protease 9(Mcpt9)	1.647125
NM_019340	regulator of G-protein signaling 3(Rgs3)	1.639602
NM_019353	thyroid peroxidase(Tpo)	1.757899
NM_019376	tyrosine 3-monooxygenase/tryptophan 5-monooxygenase activation protein, gamma(Ywhag)	1.753748
NM_019904	galectin 1(Lgals1)	1.747295
NM_020082	ribonuclease A family member 4(Rnase4)	1.5499
NM_021581	prolyl 3-hydroxylase family member 4(P3h4)	1.874377
NM_021585	mast cell immunoglobulin-like receptor 1(Milr1)	1.878356
NM_021598	mast cell protease 8(Mcpt8)	2.72646
NM_021671	transmembrane protein 33(Tmem33)	1.613433
NM_021684	adenylate cyclase 10 (soluble)(Adcy10)	1.83554
NM_021697	potassium voltage-gated channel modifier subfamily V member 1(Kcnv1)	1.756715
NM_021701	protein phosphatase 3, regulatory subunit B, beta(Ppp3r2)	1.764523
NM_021748	N-ethylmaleimide sensitive factor, vesicle fusing ATPase(Nsf)	1.720353
NM_021772	cyclin-dependent kinase-like 3(Cdkl3)	1.589505
NM_021849	RFNG O-fucosylpeptide 3-beta-N-acetylglucosaminyltransferase(Rfng)	1.75421
NM_021859	megakaryocyte-associated tyrosine kinase(Matk)	1.846828
NM_021997	CAP-GLY domain containing linker protein 2(Clip2)	1.829356
NM_022203	No data	1.914261
NM_022210	MYC associated factor X(Max)	1.814325
NM_022254	G protein-coupled receptor 85(Gpr85)	1.800299
NM_022282	discs large MAGUK scaffold protein 2(Dlg2)	1.768177
NM_022284	guanylate cyclase activator 2B(Guca2b)	2.039829
NM_022296	xylosyltransferase 2(Xylt2)	2.198775
NM_022297	dimethylarginine dimethylaminohydrolase 1(Ddah1)	1.685947
NM_022509	survival of motor neuron 1, telomeric(Smn1)	1.906114
NM_022538	phospholipid phosphatase 1(Plpp1)	1.909921
NM_022600	adenylate cyclase 5(Adcy5)	1.520435
NM_022613	No data	1.929265
NM_022623	frizzled class receptor 4(Fzd4)	2.230565
NM_022625	tropic 1808(Tpc1808)	1.653187
NM_022638	transient receptor potential cation channel, subfamily C, member 2, pseudogene(Trpc2)	1.663797
NM_022639	cholinergic receptor nicotinic alpha 10 subunit(Chrna10)	1.732239
NM_022686	histone cluster 1, H4b(Hist1h4b)	2.369333
NM_022704	mannose-binding lectin (protein C) 2(Mbl2)	1.568571
NM_022850	dipeptidyl peptidase like 6(Dpp6)	2.262092
NM_022859	cysteine-rich secretory protein 1(Crisp1)	1.609319
NM_022929	potassium voltage-gated channel interacting protein 1(Kcnip1)	1.663328
NM_022947	ClpB homolog, mitochondrial AAA ATPase chaperonin(Clpb)	1.811601
NM_022954	FAT atypical cadherin 2(Fat2)	2.727616
NM_023102	casein kinase 1, gamma 2(Csnk1g2)	1.702925
NM_023963	caudal type homeo box 2(Cdx2)	1.683684
NM_023969	lysophosphatidic acid receptor 3(Lpar3)	1.63442
NM_023973	indoleamine 2,3-dioxygenase 1(Ido1)	1.519611
NM_023981	colony stimulating factor 1(Csf1)	1.538487
NM_023994	taste receptor, type 2, member 118(Tas2r118)	1.695958
NM_023995	taste receptor, type 2, member 107(Tas2r107)	2.24126
NM_024127	growth arrest and DNA-damage-inducible, alpha(Gadd45a)	2.152067
NM_024381	glycerol kinase(Gk)	1.811678
NM_024487	GrpE-like 1, mitochondrial(Grpel1)	1.943116
NM_024489	zinc finger and BTB domain containing 10(Zbtb10)	2.056393
NM_030826	glutathione peroxidase 1(Gpx1)	1.652839
NM_030837	kidney specific organic anion transporter(Slc21a4)	2.998452
NM_030843	syntaxin binding protein 5(Stxbp5)	2.170744
NM_030860	myocyte enhancer factor 2D(Mef2d)	2.048593
NM_030868	nephroblastoma overexpressed(Nov)	1.600397
NM_030875	sodium voltage-gated channel alpha subunit 1(Scn1a)	1.622929
NM_031000	aldo-keto reductase family 1 member A1(Akr1a1)	1.567229
NM_031021	casein kinase 2 beta(Csnk2b)	1.917293
NM_031036	G protein subunit alpha q(Gnaq)	1.918023
NM_031059	msh homeobox 1(Msx1)	1.99165
NM_031065	ribosomal protein L10A(Rpl10a)	2.300643
NM_031085	protein kinase C, eta(Prkch)	2.372214
NM_031093	RAS like proto-oncogene A(Rala)	2.39697
NM_031120	signal sequence receptor, gamma(Ssr3)	1.674383
NM_031129	transcription elongation factor B subunit 2(Tceb2)	2.331149
NM_031130	nuclear receptor subfamily 2, group F, member 1(Nr2f1)	1.84239
NM_031133	thrombopoietin(Thpo)	1.757085
NM_031136	thymosin beta 4, X-linked(Tmsb4x)	2.204979
NM_031142	double C2 domain beta(Doc2b)	2.438734
NM_031240	cysteine-rich secretory protein 2(Crisp2)	2.969829
NM_031352	drebrin-like(Dbnl)	1.689691
NM_031360	sphingomyelin phosphodiesterase 2(Smpd2)	1.509688
NM_031537	RoBo-1(LOC24906)	2.382064
NM_031570	ribosomal protein S7(Rps7)	1.720451
NM_031575	AKT serine/threonine kinase 3(Akt3)	1.827906
NM_031601	calcium voltage-gated channel subunit alpha1 G(Cacna1g)	2.076481
NM_031603	tyrosine 3-monooxygenase/tryptophan 5-monooxygenase activation protein, epsilon(Ywhae)	1.516651
NM_031604	ATPase H+ transporting V0 subunit a1(Atp6v0a1)	2.734505
NM_031621	SH2B adaptor protein 3(Sh2b3)	1.87789
NM_031624	immunoglobulin (CD79A) binding protein 1(Igbp1)	1.647906
NM_031627	nuclear receptor subfamily 1, group H, member 3(Nr1h3)	1.513038
NM_031643	mitogen activated protein kinase kinase 1(Map2k1)	2.033606
NM_031728	synaptosomal-associated protein 91(Snap91)	1.989374
NM_031729	protein phosphatase 5, catalytic subunit(Ppp5c)	1.7209
NM_031762	cyclin-dependent kinase inhibitor 1B(Cdkn1b)	1.807938
NM_031781	amyloid beta precursor protein binding family A member 3(Apba3)	1.770669
NM_031789	nuclear factor, erythroid 2-like 2(Nfe2l2)	2.362231
NM_031821	polo-like kinase 2(Plk2)	2.022837
NM_031823	wolframin ER transmembrane glycoprotein(Wfs1)	2.469074
NM_031832	galectin 3(Lgals3)	1.597594
NM_031836	vascular endothelial growth factor A(Vegfa)	1.578976
NM_031974	clathrin, light chain A(Clta)	1.664435
NM_032079	DnaJ heat shock protein family (Hsp40) member A2(Dnaja2)	1.580883
NM_032082	hydroxyacid oxidase 2(Hao2)	1.813424
NM_033376	potassium two pore domain channel subfamily K member 3(Kcnk3)	1.540317
NM_053323	delta(4)-desaturase, sphingolipid 1(Degs1)	1.581652
NM_053716	fructose-bisphosphatase 2(Fbp2)	1.901898
NM_057098	transcription elongation factor A2(Tcea2)	2.570171
NM_057099	proteasome subunit beta 6(Psmb6)	1.767274
NM_078622	phosphate cytidylyltransferase 1, choline, alpha(Pcyt1a)	1.676003
NM_080900	actin filament associated protein 1(Afap1)	1.565316
NM_130420	tripartite motif-containing 9(Trim9)	2.025582
NM_130421	lymphocyte cytosolic protein 2(Lcp2)	1.912839
NM_130753	fibroblast growth factor 19(Fgf19)	1.524216
NM_133309	calpain 8(Capn8)	2.126877
NM_133321	potassium voltage-gated channel subfamily J member 15(Kcnj15)	2.331074
NM_133383	serine carboxypeptidase 1(Scpep1)	1.565457
NM_133392	serine/threonine kinase 17b(Stk17b)	2.265261
NM_133406	1-acylglycerol-3-phosphate O-acyltransferase 4(Agpat4)	1.599979
NM_133612	No data	1.653442
NM_134376	calsyntenin 3(Clstn3)	1.500037
NM_134462	ATPase secretory pathway Ca2+ transporting 2(Atp2c2)	2.49921
NM_134465	cytokine receptor-like factor 2(Crlf2)	2.519908
NM_138710	DAB2 interacting protein(Dab2ip)	1.61244
NM_139193	prolactin releasing hormone receptor(Prlhr)	1.542231
NM_144741	resistin(Retn)	1.849312
NM_144742	ATPase phospholipid transporting 11A(Atp11a)	2.002968
NM_147207	ischemia related factor vof-16(Vof16)	1.623283
U03390	receptor for activated C kinase 1(Rack1)	1.944424
U05593	Cd80 molecule(Cd80)	2.654472
U06434	C-C motif chemokine ligand 4(Ccl4)	1.819756
U14746	von Hippel-Lindau tumor suppressor(Vhl)	1.543271
U17604	reticulon 1(Rtn1)	1.825625
U18771	RAB26, member RAS oncogene family(Rab26)	1.820437
U39208	cytochrome P450, family 4, subfamily f, polypeptide 6(Cyp4f6)	1.645854
U48828	No data	1.727898
U51583	zinc finger E-box binding homeobox 1(Zeb1)	1.51051
U53475	RAB8B, member RAS oncogene family(Rab8b)	1.913337
U57063	granzyme F(Gzmf)	1.980644
U57391	SH2B adaptor protein 1(Sh2b1)	2.074809
U72353	lamin B1(Lmnb1)	2.074554
U92564	zinc finger protein 423(Zfp423)	2.090788
U93851	cyclic nucleotide gated channel alpha 1(Cnga1)	1.506566
U94856	paraoxonase 1(Pon1)	1.546413
X51992	gamma-aminobutyric acid type A receptor alpha 5 subunit(Gabra5)	1.648049
X52952	Moloney sarcoma oncogene(Mos)	1.892727
X56190	No data	1.501611
X63281	No data	2.264276
X69029	No data	1.553785
X80671	olfactory receptor 1271(Olr1271)	1.74883
X81193	cysteine and glycine rich protein 3(Csrp3)	1.681766
X89603	metallothionein 3(Mt3)	2.362925
X89962	cold shock domain containing C2(Csdc2)	2.244
X96790	glutamate metabotropic receptor 7(Grm7)	1.589366
X98490	replication protein A2(Rpa2)	1.518939
Z75029	heat shock 70kD protein 1B (mapped)(Hspa1b)	1.844737

## Appendix B. List of Genes Down-Regulated by PE Effect

**Gene Id**	**Gene**	**Z-Score**
AA944170	S/D	−1.506199
AA944489	No data	−1.530233
AA996993	No data	−2.983268
AB011529	cadherin, EGF LAG seven-pass G-type receptor 2(Celsr2)	−1.74635
AB011679	tubulin, beta 5 class I(Tubb5)	−2.325114
AB020504	No data	−2.211124
AB020757	chymotrypsin-like(Ctrl)	−2.059928
AB025017	No data	−2.29894
AB033418	No data	−2.907947
AB047540	isocitrate dehydrogenase 3 (NAD+) beta(Idh3B)	−1.980831
AB067445	integrin alpha 2(Itga2)	−1.977614
AF000944	general transcription factor IIA, 2(Gtf2a2)	−2.614055
AF006664	NK2 homeobox 5(Nkx2-5)	−1.571779
AF012714	multiple inositol-polyphosphate phosphatase 1(Minpp1)	−1.517925
AF015953	aryl hydrocarbon receptor nuclear translocator-like(Arntl)	−1.67005
AF016387	retinoid X receptor gamma(Rxrg)	−1.847981
AF021936	CDC42 binding protein kinase beta(Cdc42bpb)	−2.024961
AF026505	sorbin and SH3 domain containing 2(Sorbs2)	−1.84203
AF032120	GIPC PDZ domain containing family, member 1(Gipc1)	−1.832343
AF037071	nitric oxide synthase 1 adaptor protein(Nos1ap)	−2.242322
AF039584	CD55 molecule, decay accelerating factor for complement(Cd55)	−1.707524
AF053093	No data	−1.976489
AF053097	No data	−1.603483
AF084544	versican(Vcan)	−1.711944
AF092090	polyamine modulated factor 1 binding protein 1(Pmfbp1)	−1.629489
AF123651	spermatogenesis associated 2(Spata2)	−1.791841
AF151710	No data	−1.874231
AF170284	gap junction protein, beta 6(Gjb6)	−2.501274
AF193757	N-terminal EF-hand calcium binding protein 2(Necab2)	−1.515331
AF200359	UDP-glucose glycoprotein glucosyltransferase 1(Uggt1)	−2.250208
AF243515	BCL2/adenovirus E1B interacting protein 3(Bnip3)	−1.627503
AF247450	hyperpolarization-activated cyclic nucleotide-gated potassium channel 1(Hcn1)	−1.51455
AF277901	zinc finger protein 483(Zfp483)	−1.798414
AF302047	CXADR-like membrane protein(Clmp)	−2.140061
AF304429	mitochondrial pyruvate carrier 1(Mpc1)	−2.257792
AF307852	ARP3 actin related protein 3 homolog(Actr3)	−1.95466
AF323615	phospholipase C, epsilon 1(Plce1)	−4.175461
AF329856	phosphodiesterase 1C(Pde1c)	−1.504374
AF347935	interleukin 11(Il11)	−1.674463
AF361476	Cbp/p300-interacting transactivator, with Glu/Asp-rich carboxy-terminal domain, 2(Cited2)	−1.710103
AF367467	selenoprotein S(Selenos)	−2.382484
AF379608	doublesex and mab-3 related transcription factor 1(Dmrt1)	−1.683129
AF400662	calcium voltage-gated channel auxiliary subunit alpha2delta 1(Cacna2d1)	−1.543195
AF537333	aurora kinase A(Aurka)	−1.661243
AI059699	No data	−1.842174
AI136886	No data	−1.650746
AI229387	No data	−1.511607
AI598392	No data	−1.725522
AJ006855	synaptojanin 1(Synj1)	−1.727807
AJ131196	No data	−1.769069
AJ250280	potassium voltage-gated channel subfamily H member 5(Kcnh5)	−1.520944
AJ303374	ATP binding cassette subfamily G member 1(Abcg1)	−1.609867
AJ409332	TIMP metallopeptidase inhibitor 2(Timp2)	−2.3002
AW141281	No data	−2.051067
AW141994	No data	−1.692865
AW434257	No data	−1.789087
AW915590	No data	−1.557293
AW916146	No data	−1.959788
AW916635	No data	−1.746735
AW917632	No data	−1.549931
AW918748	No data	−2.166549
AW918775	No data	−1.649553
AW919008	No data	−1.692303
AY011335	No data	−1.711045
AY014898	inositol polyphosphate multikinase(Ipmk)	−2.342213
AY026512	dynein light chain roadblock-type 1(Dynlrb1)	−2.119183
BF284301	No data	−2.121369
BF287132	No data	−2.068833
BF392368	No data	−2.212956
BF395339	No data	−1.928218
BF399588	No data	−2.70933
BF414052	No data	−2.155561
BF416262	No data	−1.546722
BF524417	No data	−2.049708
BF549771	No data	−1.690628
BF555199	No data	−1.523037
BF559446	No data	−2.697068
BF560218	No data	−2.252126
BF566173	No data	−2.261156
BF567456	No data	−1.898923
BG663098	No data	−1.5361
BG664103	No data	−2.448298
BG666505	No data	−1.572982
BG667467	No data	−2.508352
BG671325	No data	−1.655659
BI278738	No data	−2.62235
BI295378	No data	−2.278025
BI296499	No data	−2.01441
BM386847	No data	−2.077829
BQ194714	No data	−1.88522
BQ205274	NADH:ubiquinone oxidoreductase subunit A12(Ndufa12)	−2.38962
BQ208291	No data	−1.636124
BU670896	No data	−3.513474
BU671010	No data	−2.556612
BU671095	No data	−2.578096
BU671151	No data	−1.744558
D00920	seminal vesicle secretory protein 3A(Svs3a)	−2.000729
D14048	heterogeneous nuclear ribonucleoprotein U(Hnrnpu)	−2.261341
D16465	adenylate cyclase activating polypeptide 1 receptor type 1(Adcyap1r1)	−1.966142
D16479	hydroxyacyl-CoA dehydrogenase/3-ketoacyl-CoA thiolase/enoyl-CoA hydratase (trifunctional protein), beta subunit(Hadhb)	−1.944151
D26178	intestinal cell kinase(Ick)	−2.540983
D63772	solute carrier family 1 member 1(Slc1a1)	−1.665296
J02585	stearoyl-CoA desaturase(Scd)	−2.299181
J02868	cytochrome P450, family 2, subfamily d, polypeptide 3(Cyp2d3)	−1.688779
J04628	3-hydroxyisobutyrate dehydrogenase(Hibadh)	−1.717486
L02121	cyclin-dependent kinase 5(Cdk5)	−2.181096
L02315	calcium voltage-gated channel auxiliary subunit beta 4(Cacnb4)	−1.528497
L10362	synaptic vesicle glycoprotein 2b(Sv2b)	−1.569646
L12407	No data	−1.867835
L22654	gamma-2a immunoglobulin heavy chain(IgG-2a)	−1.709121
L27487	calcitonin receptor-like(Calcrl)	−1.970351
M11794	No data	−1.536746
M15202	No data	−1.947036
M15327	alcohol dehydrogenase 1 (class I)(Adh1)	−2.079242
M15481	insulin-like growth factor 1(Igf1)	−2.298139
M16409	cholinergic receptor, muscarinic 4(Chrm4)	−1.743017
M18841	No data	−1.998377
M29295	small nuclear ribonucleoprotein polypeptides B and B1(Snrpb)	−1.675114
M30692	No data	−2.027642
M31322	amyloid beta precursor like protein 2(Aplp2)	−1.814887
M57705	thyroid peroxidase(Tpo)	−1.513088
M61142	thimet oligopeptidase 1(Thop1)	−1.696997
M63837	platelet derived growth factor receptor alpha(Pdgfra)	−1.822324
M64381	olfactory receptor 1082(Olr1082)	−1.761637
M76733	glutathione peroxidase 6(Gpx6)	−1.971279
M83143	ST6 beta-galactoside alpha-2,6-sialyltransferase 1(St6gal1)	−1.602127
M83209	BPI fold containing family A, member 2(Bpifa2)	−2.386439
M83210	BPI fold containing family A, member 2F(Bpifa2f)	−1.615422
M83745	proprotein convertase subtilisin/kexin type 1(Pcsk1)	−1.961893
M94043	RAB38, member RAS oncogene family(Rab38)	−1.660598
NM_012490	acrosin(Acr)	−1.761651
NM_012502	androgen receptor(Ar)	−2.367953
NM_012542	cytochrome P450, family 2, subfamily a, polypeptide 3(Cyp2a3)	−1.563632
NM_012589	interleukin 6(Il6)	−2.279895
NM_012595	lactate dehydrogenase B(Ldhb)	−1.56024
NM_012621	6-phosphofructo-2-kinase/fructose-2,6-biphosphatase 1(Pfkfb1)	−2.130949
NM_012635	protease, serine 1(Prss1)	−1.938556
NM_012657	serine (or cysteine) proteinase inhibitor, clade A, member 3C(Serpina3c)	−1.73983
NM_012679	No data	−1.660105
NM_012681	transthyretin(Ttr)	−1.632171
NM_012682	uncoupling protein 1(Ucp1)	−1.956669
NM_012703	thyroid hormone responsive(Thrsp)	−2.132157
NM_012706	gastrin releasing peptide receptor(Grpr)	−2.32707
NM_012740	tyrosine hydroxylase(Th)	−1.687967
NM_012777	apolipoprotein D(Apod)	−2.293886
NM_012805	retinoid X receptor alpha(Rxra)	−1.788029
NM_012825	aquaporin 4(Aqp4)	−1.738027
NM_012826	alpha-2-glycoprotein 1, zinc-binding(Azgp1)	−1.917567
NM_012841	DCC netrin 1 receptor(Dcc)	−2.060894
NM_012843	epithelial membrane protein 1(Emp1)	−1.524748
NM_012922	caspase 3(Casp3)	−1.730399
NM_012940	cytochrome P450, family 1, subfamily b, polypeptide 1(Cyp1b1)	−1.825438
NM_012949	enolase 3(Eno3)	−2.250452
NM_012951	fibroblast growth factor 10(Fgf10)	−1.581501
NM_012967	intercellular adhesion molecule 1(Icam1)	−1.527629
NM_012971	potassium voltage-gated channel subfamily A member 4(Kcna4)	−2.126831
NM_013023	S-antigen visual arrestin(Sag)	−1.921779
NM_013054	No data	−1.571264
NM_013095	SMAD family member 3(Smad3)	−1.508985
NM_013159	insulin degrading enzyme(Ide)	−1.663445
NM_013161	pancreatic lipase(Pnlip)	−2.292426
NM_013167	uncoupling protein 3(Ucp3)	−2.046956
NM_013168	hydroxymethylbilane synthase(Hmbs)	−4.18491
NM_013192	potassium voltage-gated channel subfamily J member 6(Kcnj6)	−1.544981
NM_016998	carboxypeptidase A1(Cpa1)	−1.72664
NM_017006	glucose-6-phosphate dehydrogenase(G6pd)	−2.105296
NM_017011	glutamate receptor, metabotropic 1(Grm1)	−1.945417
NM_017014	glutathione S-transferase mu 1(Gstm1)	−2.433956
NM_017019	interleukin 1 alpha(Il1a)	−1.572188
NM_017069	gamma-aminobutyric acid type A receptor alpha3 subunit(Gabra3)	−3.176886
NM_017075	acetyl-CoA acetyltransferase 1(Acat1)	−1.693916
NM_017084	glycine N-methyltransferase(Gnmt)	−1.587023
NM_017085	cytochrome P450, family 19, subfamily a, polypeptide 1(Cyp19a1)	−3.202593
NM_017093	AKT serine/threonine kinase 2(Akt2)	−1.708931
NM_017101	peptidylprolyl isomerase A (cyclophilin A)(Ppia)	−1.962416
NM_017106	chloride voltage-gated channel 5(Clcn5)	−2.303484
NM_017120	casein beta(Csn2)	−1.753558
NM_017156	cytochrome P450, family 2, subfamily b, polypeptide 12(Cyp2b12)	−1.675282
NM_017158	cytochrome P450, family 2, subfamily c, polypeptide 7(Cyp2c7)	−1.9999
NM_017177	choline kinase beta(Chkb)	−1.598296
NM_017179	UNC homeobox(Uncx)	−1.985105
NM_017190	myelin-associated glycoprotein(Mag)	−1.957495
NM_017197	CUGBP, Elav-like family member 2(Celf2)	−2.358172
NM_017228	atrophin 1(Atn1)	−3.12621
NM_017265	hydroxy-delta-5-steroid dehydrogenase, 3 beta- and steroid delta-isomerase 6(Hsd3b6)	−1.512438
NM_017285	proteasome subunit beta 3(Psmb3)	−2.192897
NM_017291	gamma-aminobutyric acid type A receptor rho 1 subunit(Gabrr1)	−3.620853
NM_017292	gamma-aminobutyric acid type A receptor rho 2 subunit(Gabrr2)	−1.576548
NM_017326	calmodulin 2(Calm2)	−2.141575
NM_017337	phosphodiesterase 3A(Pde3a)	−1.72263
NM_017339	ISL LIM homeobox 1(Isl1)	−2.983281
NM_017343	myosin light chain 12B(Myl12b)	−1.748003
NM_019182	ring finger protein 4(Rnf4)	−1.852677
NM_019190	CD46 molecule(Cd46)	−1.945158
NM_019211	RAS guanyl releasing protein 1(Rasgrp1)	−1.760087
NM_019241	gap junction protein, beta 5(Gjb5)	−1.748274
NM_019248	neurotrophic receptor tyrosine kinase 3(Ntrk3)	−1.880549
NM_019255	calcium voltage-gated channel auxiliary subunit gamma 1(Cacng1)	−1.569839
NM_019261	killer cell lectin-like receptor subfamily C, member 2(Klrc2)	−2.129231
NM_019270	potassium voltage-gated channel subfamily A member 3(Kcna3)	−2.943964
NM_019278	regulated endocrine-specific protein 18(Resp18)	−2.015828
NM_019285	adenylate cyclase 4(Adcy4)	−1.51557
NM_019299	clathrin heavy chain(Cltc)	−1.670579
NM_019348	somatostatin receptor 2(Sstr2)	−1.921952
NM_019354	uncoupling protein 2(Ucp2)	−2.271891
NM_019363	aldehyde oxidase 1(Aox1)	−1.50887
NM_019620	zinc finger protein 386 (Kruppel-like)(Zfp386)	−2.6169
NM_019621	discs large MAGUK scaffold protein 4(Dlg4)	−1.768061
NM_019907	CXXC repeat containing interactor of PDZ3 domain(Cript)	−1.965978
NM_020074	serglycin(Srgn)	−1.792037
NM_020076	3-hydroxyanthranilate 3,4-dioxygenase(Haao)	−1.647168
NM_020096	interferon-induced protein with tetratricopeptide repeats 1(Ifit1)	−1.569029
NM_020301	ADAM metallopeptidase domain 7(Adam7)	−1.648235
NM_020542	C-C motif chemokine receptor 1(Ccr1)	−1.713195
NM_021580	prolactin family 8, subfamily a, member 4(Prl8a4)	−2.141944
NM_021659	synaptotagmin 7(Syt7)	−1.795637
NM_021672	growth differentiation factor 9(Gdf9)	−1.566303
NM_021676	SH3 and multiple ankyrin repeat domains 3(Shank3)	−2.237337
NM_021703	A-kinase anchoring protein 14(Akap14)	−2.557232
NM_021742	nuclear receptor subfamily 5, group A, member 2(Nr5a2)	−1.603742
NM_021769	sulfotransferase family 1D, member 1(Sult1d1)	−1.883171
NM_022178	myosin VA(Myo5a)	−2.420753
NM_022196	leukemia inhibitory factor(Lif)	−1.875411
NM_022202	glutamate metabotropic receptor 8(Grm8)	−1.511956
NM_022204	exocyst complex component 5(Exoc5)	−1.572194
NM_022214	C-X-C motif chemokine ligand 6(Cxcl6)	−2.525867
NM_022241	prostaglandin D2 receptor(Ptgdrl)	−1.557255
NM_022261	B-box and SPRY domain containing(Bspry)	−1.56046
NM_022278	glutaredoxin(Glrx)	−2.208115
NM_022283	No data	−2.543935
NM_022386	MAF bZIP transcription factor G(Mafg)	−2.383068
NM_022388	FXYD domain-containing ion transport regulator 4(Fxyd4)	−2.897865
NM_022394	scaffold attachment factor B(Safb)	−2.659302
NM_022502	palmitoyl-protein thioesterase 1(Ppt1)	−1.90058
NM_022503	cytochrome c oxidase subunit VIIa polypeptide 2(Cox7a2)	−1.523241
NM_022504	ribosomal protein L36(Rpl36)	−1.906632
NM_022506	ribosomal protein L31(Rpl31)	−2.087805
NM_022530	prolactin family 7, subfamily a, member 3(Prl7a3)	−1.576727
NM_022595	PDGFA associated protein 1(Pdap1)	−2.240738
NM_022597	cathepsin B(Ctsb)	−2.37573
NM_022612	BCL2 like 11(Bcl2l11)	−1.597404
NM_022634	leukocyte specific transcript 1(Lst1)	−1.756651
NM_022694	staphylococcal nuclease and tudor domain containing 1(Snd1)	−2.119255
NM_022711	steroid 5 alpha-reductase 2(Srd5a2)	−1.557239
NM_022797	glutamate ionotropic receptor NMDA type subunit 2D(Grin2d)	−2.63892
NM_022863	iron responsive element binding protein 2(Ireb2)	−1.923661
NM_022867	microtubule-associated protein 1 light chain 3 beta(Map1lc3b)	−1.780024
NM_022949	ribosomal protein L14(Rpl14)	−1.533009
NM_023095	mannosyl (alpha-1,6-)-glycoprotein beta-1,6-N-acetyl-glucosaminyltransferase(Mgat5)	−2.000325
NM_023100	neuromedin U receptor 1(Nmur1)	−1.627031
NM_023974	synaptoporin(Synpr)	−1.803122
NM_023998	taste receptor, type 2, member 13(Tas2r13)	−1.675877
NM_024156	annexin A6(Anxa6)	−1.687137
NM_024346	stathmin 3(Stmn3)	−1.575717
NM_024360	hes family bHLH transcription factor 1(Hes1)	−2.146919
NM_024385	hematopoietically expressed homeobox(Hhex)	−1.692521
NM_024388	nuclear receptor subfamily 4, group A, member 1(Nr4a1)	−2.520533
NM_024397	abl-interactor 1(Abi1)	−1.624808
NM_024404	heterogeneous nuclear ribonucleoprotein D(Hnrnpd)	−3.042042
NM_024483	adrenoceptor alpha 1D(Adra1d)	−1.736564
NM_024484	5′-aminolevulinate synthase 1(Alas1)	−1.714727
NM_030857	LYN proto-oncogene, Src family tyrosine kinase(Lyn)	−2.18526
NM_031008	adaptor-related protein complex 2, alpha 2 subunit(Ap2a2)	−1.552738
NM_031055	matrix metallopeptidase 9(Mmp9)	−2.852522
NM_031057	aldehyde dehydrogenase 6 family, member A1(Aldh6a1)	−1.519431
NM_031088	prostaglandin E receptor 2(Ptger2)	−2.221709
NM_031127	sulfite oxidase(Suox)	−2.397507
NM_031236	fucosyltransferase 1(Fut1)	−1.985466
NM_031327	cysteine-rich, angiogenic inducer, 61(Cyr61)	−1.638485
NM_031346	polypyrimidine tract binding protein 3(Ptbp3)	−1.590357
NM_031531	serine (or cysteine) peptidase inhibitor, clade A, member 3N(Serpina3n)	−2.119708
NM_031549	transgelin(Tagln)	−2.37869
NM_031563	Y box binding protein 1(Ybx1)	−1.584841
NM_031590	WNT1 inducible signaling pathway protein 2(Wisp2)	−1.877269
NM_031678	period circadian clock 2(Per2)	−1.587107
NM_031706	ribosomal protein S8(Rps8)	−2.051886
NM_031719	chloride nucleotide-sensitive channel 1A(Clns1a)	−1.549741
NM_031720	deiodinase, iodothyronine, type II(Dio2)	−1.958028
NM_031786	tripartite motif-containing 3(Trim3)	−1.668096
NM_031792	sperm associated antigen 4(Spag4)	−1.634627
NM_031800	death effector domain-containing(Dedd)	−1.641957
NM_031808	calpain 6(Capn6)	−1.820196
NM_031810	defensin beta 1(Defb1)	−2.054222
NM_031828	potassium calcium-activated channel subfamily M alpha 1(Kcnma1)	−1.606975
NM_032056	transporter 2, ATP binding cassette subfamily B member(Tap2)	−2.753854
NM_032062	kalirin, RhoGEF kinase(Kalrn)	−1.810885
NM_033021	SEC31 homolog A, COPII coat complex component(Sec31a)	−1.750599
NM_033095	crystallin, gamma D(Crygd)	−1.569465
NM_053649	kringle containing transmembrane protein 1(Kremen1)	−1.821446
NM_053674	phytanoyl-CoA 2-hydroxylase(Phyh)	−1.905711
NM_053701	calcium voltage-gated channel subunit alpha1 F(Cacna1f)	−1.62128
NM_080689	dynamin 1(Dnm1)	−1.617324
NM_080783	UDP-galactose-4-epimerase(Gale)	−2.373817
NM_130405	KH RNA binding domain containing, signal transduction associated 1(Khdrbs1)	−1.779596
NM_131914	caveolin 2(Cav2)	−1.636241
NM_133403	E1A binding protein p300(Ep300)	−2.567324
NM_133594	small ubiquitin-like modifier 2(Sumo2)	−1.569797
NM_134375	NLR family, pyrin domain containing 6(Nlrp6)	−2.192127
NM_134387	dicarbonyl and L-xylulose reductase(Dcxr)	−2.210838
NM_134432	angiotensinogen(Agt)	−1.737263
NM_134457	siah E3 ubiquitin protein ligase 2(Siah2)	−1.625673
NM_138851	prokineticin 1(Prok1)	−1.918202
NM_138890	EH-domain containing 3(Ehd3)	−1.507669
NM_138976	mitofusin 1(Mfn1)	−1.671108
NM_139082	BMP and activin membrane-bound inhibitor(Bambi)	−2.450906
NM_139097	sodium voltage-gated channel beta subunit 3(Scn3b)	−3.059727
NM_139258	Bcl2 modifying factor(Bmf)	−2.168055
NM_139336	UDP-glucuronate decarboxylase 1(Uxs1)	−1.54412
NM_145673	MAF bZIP transcription factor K(Mafk)	−1.536959
NM_147137	cystatin SC(LOC257643)	−3.032693
NM_152849	homeobox and leucine zipper encoding(Homez)	−1.725448
S75437	No data	−1.965821
U03407	proline rich, lacrimal 1(Prol1)	−2.285635
U03417	olfactomedin 1(Olfm1)	−1.797439
U03630	store-operated calcium entry-associated regulatory factor(Saraf)	−2.016451
U20286	torsin 1A interacting protein 1(Tor1aip1)	−1.727547
U21954	Eph receptor A7(Epha7)	−1.630146
U22663	No data	−1.993711
U24489	tenascin XA, pseudogene 1(Tnxa-ps1)	−2.172308
U28356	protein tyrosine phosphatase, non-receptor type 7(Ptpn7)	−2.374227
U35371	contactin 4(Cntn4)	−1.667741
U40652	protein tyrosine phosphatase, receptor type, N(Ptprn)	−1.867797
U41663	neuroligin 3(Nlgn3)	−1.591787
U41803	mitofusin 2(Mfn2)	−1.574964
U41853	hypoxia up-regulated 1(Hyou1)	−1.657766
U44129	lectin, mannose-binding, 1(Lman1)	−2.451062
U49694	acyl-CoA thioesterase 7(Acot7)	−1.924287
U50948	No data	−1.881066
U53420	No data	−4.016245
U56241	MAF bZIP transcription factor B(Mafb)	−2.421294
U56936	killer cell lectin-like receptor subfamily B member 1B(Klrb1b)	−1.830188
U57362	collagen type XII alpha 1 chain(Col12a1)	−2.0687
U62316	solute carrier family 16 member 7(Slc16a7)	−2.847701
U69702	activin A receptor type 1C(Acvr1c)	−1.705311
U76206	purinergic receptor P2Y14(P2ry14)	−2.320753
U78304	cartilage acidic protein 1(Crtac1)	−1.778874
U81037	neuronal cell adhesion molecule(Nrcam)	−1.502983
U92010	similar to RIKEN cDNA D230025D16Rik(RGD621098)	−1.975117
V01222	albumin(Alb)	−2.265804
X01153	No data	−1.843423
X05034	No data	−1.718179
X13309	WAP four-disulfide core domain 18(Wfdc18)	−2.535182
X53003	acetyl-CoA carboxylase alpha(Acaca)	−1.756607
X54549	transcription factor 3(Tcf3)	−2.43995
X56328	No data	−2.636174
X60822	methionine adenosyltransferase 1A(Mat1a)	−1.586548
X64411	ubiquitin protein ligase E3 component n-recognin 5(Ubr5)	−2.174631
X68101	dedicator of cytokinesis 9(Dock9)	−4.49773
X71463	No data	−1.940264
X73579	Fc fragment of IgE receptor II(Fcer2)	−1.640805
X74815	myosin IE(Myo1e)	−1.973621
X78167	ribosomal protein L15(Rpl15)	−2.28316
X78604	ADP-ribosylation factor like GTPase 5A(Arl5a)	−1.523338
X84004	dual specificity phosphatase 1(Dusp1)	−2.325959
Y12178	ceruloplasmin(Cp)	−1.709408
Y17319	No data	−1.766901
Y17325	NSA2 ribosome biogenesis homolog(Nsa2)	−1.702431
Y17328	crystallin, mu(Crym)	−1.788589
